# Urban Human–Coyote Conflicts: Assessing Friendliness as an Indicator of Coexistence

**DOI:** 10.3390/ani13182903

**Published:** 2023-09-13

**Authors:** Cameron T. Whitley, Melanie M. Bowers, Harriett Grantz

**Affiliations:** 1 Department of Sociology, Western Washington University, Bellingham, WA 98225, USA; grantzh@wwu.edu; 2 Department of Political Science, Western Washington University, Bellingham, WA 98225, USA; whitlem@wwu.edu

**Keywords:** human–animal conflict, urban coyotes, survival of the friendliest, human–animal studies, urban wildlife, wildlife management plans

## Abstract

**Simple Summary:**

Coyotes live in most major cities across North and Central America. As their habitat shrinks, human–coyote interactions increase, spurring debate about how to respond. Residents often fear coyotes and want extermination, but scientists argue they are a permanent fixture that play a vital ecosystem role and that eradicating them does not work and has negative impacts. Instead, ecologists argue that residents need to change their behavior to coexist with coyotes. Whether the public supports coexistence plans stems from public perceptions of coyotes, which are influenced by affective feelings, personal beliefs and experiences, and the media. Consequently, understanding how the media discusses these issues, whether they frame coyotes as friend or foe, and whether they cover coexistence is essential. To do this, we analyze coyote news coverage from 2000 to 2022 in three US cities: Los Angeles, CA; Seattle, WA; and Boston, MA. We find that the media uses friendly language to discuss coexistence and unfriendly language to justify eradication. Cities vary in the degree to which their coverage aligns with the scientific consensus; this likely reflects historical, cultural, and political views of coyotes. In Los Angeles, where coyote conflict is long-running and political, the coverage is the most unfriendly and is significantly more likely to include discussion of eradication; in Seattle and Boston, where coyotes have more recently become an issue, the coverage is far more likely to focus on coexistence. We argue that if governments want increased support for coexistence, there must be efforts to both account for media coverage in management plans and to educate the media on scientific consensus and the ecological implications of lethal management.

**Abstract:**

Human–coyote sightings and interactions are becoming more frequent in urban areas across North and Central America. While many species have lost territory, the coyote range has expanded. Relatively recently, ecologists have coalesced around the idea that coexistence is the most promising avenue to reduce human–coyote conflict in urban areas. Despite this, calls for the eradication of coyotes continue. We apply and extend the theory of survival of the friendliest to evaluate how the media is framing coyotes and management strategies and what the implications of this framing might be. Through a content analysis of newspaper articles from three different urban areas in the US (Los Angeles, CA; Seattle, WA; and Boston, MA), from 2000 to 2022, we find that friendly language is used to promote coexistence, while unfriendly language (threat, hostile, unfriendly, and danger) is used to justify eradication. We also find considerable variation in the type of coverage and consistency with scientific consensus across cities, likely reflecting the cities’ varied histories and cultural understandings of the species. Given the media’s influence on the public’s views of coyotes and their support for management strategies, these findings suggest that the media plays a central role in shaping coyote–human relationships and management strategies.

## 1. Introduction

In 2022, Nahant, a small Massachusetts town, became the first in the state to hire the federal government to eradicate its local coyote pack after it was reported that the pack of eight to ten coyotes was not friendly but a dangerous nuisance that had killed pets [1]. Animal advocates, such as the Massachusetts Society for the Prevention of Cruelty to Animals, vocalized their disdain for the idea, arguing that simply killing the pack would not deter future inhabitation and that the city needed to consider a comprehensive plan for addressing human–coyote conflict [1]. Considering the impacts of urban coyotes have become an issue for many major metropolitan areas across the US, Canada, Mexico, and Central America, as human–wildlife conflicts become more frequent [2].

Although there is no comprehensive database tracking coyote interactions with humans or pets, anecdotal evidence of rising human–coyote interactions abound, which has prompted the development of a variety of municipal, university, and nonprofit efforts to assess the issue. For example, in 2000, Cook County, IL, developed the Urban Coyote Research Project to better understand coyote ecology in the Chicago metro area [3], and in Puget Sound, Seattle University and the Woodland Park Zoo have partnered to develop the Seattle Urban Carnivore Project [4]. Documenting over 1100 human–coyote interactions in Seattle over the last year alone, efforts like the Carnivore Counter, a website where Seattleites can report sightings and interactions, demonstrate the reality that human–coyote interactions in urban environments are enduring now and for the foreseeable future. These and other similar efforts have a goal not only of understanding coyote urban ecology but of promoting human–coyote coexistence through public education and efforts to decrease human–coyote conflict. Many ecologists consider coexistence and conflict mitigation to be the only sustainable response to urban coyotes, since efforts to eradicate them have not only failed but often create larger problems [5,6,7,8,9]. Despite this, scientists have limited direct influence on public perceptions. Instead, public opinion reflects individuals’ affective feelings, fear, and beliefs about the species [6,7,8,9,10,11,12,13], which are shaped by personal experience and knowledge [14,15], social norms—particularly in their interaction with demographic characteristics [15,16]—and the media.

Because of their central role in framing and priming the public on key social and policy issues like human–coyote interactions [17], the media has significant influence over the public narrative and whether coyotes are viewed as friends or foes [18,19,20]. This type of media coverage can significantly impact affective feelings, beliefs, and support for management policies and, thus, it is essential to understand how the media portrays coyotes in urban environments and whether their portrayals align with the coexistence approach most ecologists advocate. 

Of particular importance is understanding whether the media portrays coyotes as aggressive, neutral, or friendly. Headlines that sensationalize and vilify coyotes because of attacks, for example, are likely to draw attention and negatively influence perceptions, since negative news increases negative affective feelings [21] and, for some, increases the likelihood of remembering the event [22]; but it is essential to understand how common these types of stories are, since, though deeply traumatic for those involved, negative human–coyote interactions and actual attacks by coyotes are rare. For example, of the 1100–1300 annual human–coyote interactions in Chicago [23], which can include observations of coyote activity, sightings, direct encounters, or human and pet attacks, the Urban Coyote Research Project documents only approximately a dozen attacks on dogs each year and has only identified a handful of attacks on humans over the last decade [3]. When there are conflicts, they typically stem from five sources: population and urban expansion in housing, infrastructure changes, agriculture alterations, habitat loss, and climate change [24]. In many urban environments, these things are occurring at a rapid and ongoing pace, dramatically increasing human–coyote interactions, the likelihood of media coverage, and the need for the public management of human–coyote relationships. 

In general, wildlife is managed at the state and federal levels, but with coyotes and other carnivores more regularly appearing in cities, local governments are called to respond. Municipal management strategies must address a community’s views on coyotes and, because of this, do not always rely on scientific analysis, but instead reflect the community’s norms, values, and beliefs about coyotes [1]. The implication is that local decisions to coexist, deter, relocate, or destroy “problem” animals are often driven by public perceptions of the animal and by vocal community members’ beliefs [24]. When localities do pursue scientifically driven plans, it is often in opposition to public pressure and requires significant public justification and education, as was the case in Belvedere, CA. In the face of calls to hire snipers to eliminate the local coyote population, Belvedere’s City Council passed a scientifically grounded coyote management plan focused on coexistence. Doing so was met with community opposition, and the city had to send letters and develop an educational website to help explain the policy and overcome public disdain [25]. While local government and organizations certainly play an important role in educating the public and influencing perceptions, the media is also critical in shaping views of coyotes and management strategies. 

Years of research have demonstrated that the way media outlets depict animal life dramatically influences the public’s view of species [26], with negative depictions and mediated encounters in entertainment venues reducing human–animal connections [27] and potentially inhibiting conservation behaviors [28]. Perhaps predictably, negative perceptions are strongly correlated with media reports depicting wolves as unfriendly and reintroduction as a danger to humans, children, pets, hunting opportunities, livestock, and livelihoods [29]. These findings demonstrate how important media depictions are in shaping support and opposition for scientifically driven ecological policy and the degree to which altered media depictions and public outreach are needed to reshape public perceptions in ways that promote coexistence and conservation. How this happens and what messaging is most effective is part of emerging research, but there are both theoretical and empirical reasons to think that friendliness (or lack thereof) is a key part of media framing both in terms of encouraging support for scientifically sound policy and for our broader success in an ever-changing urban human–animal landscape. 

In this context, friendliness connotes a species’ disposition towards goodwill, warmth, and kindness; understanding a species as friendly (and being friendly back) may be key to our ability to coexist from a policy and evolutionary standpoint. While evolutionary theory and popular understanding have largely focused on survival of the fittest, which highlights strength as the key component of survival, Hare and Woods [30,31,32] expand this idea in evolutionary biology with what they call survival of the friendliest. This theoretical framework suggests that human evolution occurred not simply because of ferocity but because of friendliness and cooperation. Though the authors largely focus on human–human friendliness, they also discuss the importance of this process for human relationships with nonhuman animals, where learning to work and coexist with nonhuman animal life has been essential for everything from the development of agriculture to the expansion of human territory. We discuss this theory extensively below and then use it as a lens to evaluate media depictions of species friendliness and the implications this might have for coyotes, human–coyote interactions, and urban policy, starting with the central theoretical claim that whether we identify and depict our interactions with wild animals as friendly or unfriendly influences public perceptions and willingness to coexist or demand the eradication of a species. This is novel in that few studies have applied this theoretical framework, and none have done so within the social sciences. 

In this study, we analyzed major newspaper depictions of coyotes from 2000 to 2022 in three US cities chosen for their diverse geography and history with coyotes: Los Angeles, CA; Seattle, WA; and Boston, MA. We specifically assessed how friendliness and cooperation is discussed in the media coverage of human–coyote encounters and whether friendliness or the lack thereof is used to justify management efforts (either coexistence or species removal). We focus on media coverage of coyotes (*Canis latrans*) as opposed to other predators for four reasons. First, studies on human and coyote interactions and conflict are limited [33,34]. Second, while other predators have faced diminished range and are increasingly threatened, coyotes have expanded their range to include virtually all of North and Central America, and they are expected to continue to expand their territory [35]. Third, coyotes serve an important function in our ecosystems, such as regulating smaller species, like rodents, skunks, raccoons, and foxes, and it is thus imperative that urban localities learn to coexist. Fourth, the road to coexistence may be more challenging for coyotes, because they have long been labeled as tricksters, jokers, and shapeshifters, which presents a particular challenge for communities interested in promoting coexistence and highlights the need to understand the way media portrays these animals [36,37]. By analyzing media portrayals of friendliness, we seek to apply and extend the theory of survival of the friendliest in a social science and policy context.

## 2. Background and Theory

### 2.1. State and National Policy around Coyotes

Because of their liminal status, coyotes have limited protections in the US. Coyote hunting in rural environments is common, as is euthanizing “problem” animals in urban environments [38]. While other predators like bears have limits on how many individuals can be killed in a season, there are no limits on coyote hunting. State hunting laws vary, but in most states, such as Washington, the Department of Fish and Wildlife do not classify coyotes as game animals, which means a state license *is* required to hunt and trap them. However, license enforcement for coyote removal is challenging, because individuals can kill or trap coyotes without a license if they pose a threat to the property owner’s immediate family, employee, tenant, crops, or domestic animals [39]. 

Researchers estimate that approximately half a million coyotes are trapped, poisoned, or shot each year by federal agencies, with most killed by the USDA Wildlife Services division with the stated purpose of protecting livestock [38]. Although urban coyotes are not killed at the same rate as those in rural or agricultural areas, calls for eradication have mounted in towns and cities, like Nahant and Belvedere, where residents believe the coyote population poses a significant threat. Importantly though, evolving research suggests that all methods of eradicating coyotes have largely failed, with many having unintended consequences, such as the disruption of hierarchies within packs leading to increased reproduction rates [40]. 

### 2.2. Urban Coyotes

Over the last several hundred years coyotes have expanded their traditional range from the central US and Mexican prairies to the entire continental United States, Alaska, Canada, and into Central America; today, they have a presence in virtually every US city [41,42]. Although seeing signs of coyotes can be unnerving for urban populations, the species is considered a nuisance primarily because of the perceived risk of attack rather than infestation, disease, or property damage that lead other species to be considered pests. Yet, despite their dramatically increased range, coyote attacks on humans and pets remain rare [34,43]. In part, this is because coyotes are considered synanthropic animals that adapt to coexist with humans. For example, research shows that rural coyotes are active both day and night but urban coyotes dramatically reduce their daytime activity to avoid humans [44]. Because of this adaptation, as coyote populations increase, human–coyote encounters and attacks remain relatively low. Despite this, perhaps the single greatest reason that attacks still occur is that even in urban environments, coyotes need natural or semi-natural spaces where they can live without human interference; when these areas decrease, such as when cities expand into previously wild areas, communities experience more encounters and attacks. For example, when building density increased and forested areas decreased in Charlotte, North Carolina, human–coyote encounters increased [45]. In general, coyote attacks on humans are more common in areas with higher rates of development and less forest cover, with highly developed areas in the Western US experiencing more attacks than other areas [46]. 

Importantly, given the fact that coyotes continue to expand their range and have largely been immune to eradication efforts, many ecologists now believe the greatest deterrent urban populations have to coyote encounters and attacks is behavioral change among humans [5]. Human actions, like taking dogs on off-leash walks at dusk or leaving food out for wildlife, often precipitate attacks. In fact, in one study, in one-third of cases where coyotes attacked, humans had previously been feeding them in the area [47]. As a result, there are increasing pressures to develop human-centered management plans that focus on things like keeping trash in closed containers, avoiding coyote habitats at dawn and dusk, and ensuring that pets are supervised and/or on leash. Plans focused on coexistence are emerging in cities throughout the United States and Canada but often require extensive work to address negative public perceptions and fear. A study out of Cape Town, South Africa, demonstrates that as much as 60 percent of the public’s tolerance of a species in an urban environment reflects their estimation of the risks and benefits the species brings [48]; as a result, municipal education efforts are often focused on highlighting coyotes’ potential benefits—like reducing the rodent population—while educating the public on how to reduce the risks and even transferring some of the causal story about the risk’s existence to human action. For example, when Toronto launched the Coyote Response Strategy, which focuses on coexistence and human behavioral change, it held community meetings, developed park signage, and created a website to educate the public about coyotes and the plan [41] while also making clear that the public was responsible for protecting their families and pets from their coyote cohabitants in common spaces [49]. 

### 2.3. Perceptions of Urban Coyotes

Although studies assessing the public’s view of coyotes are limited, recent research emphasizes how important beliefs and affective feelings are in shaping perspectives of the species. In their study of Newfoundlanders, Frank and colleagues [50] identified several key beliefs that drive negative views of coyotes, namely, that coyotes do not have a right to exist, are unimportant for future generations, and significantly impact daily life. Similarly, Drake and colleagues [33] analyzed public views of coyotes across four North Carolina cities to assess how place and personal characteristics influenced perceptions of danger, views on living near coyotes, and support for lethal removal. The strongest predictor of support for coyotes and opposition to lethal removal were respondents’ affective connections, which are positive emotional bonds with nature and a broad understanding of the self within the context of nature [51]. One of Drake and colleagues’ [33] most important findings was that whether respondents’ were knowledgeable about coyotes biological traits or not made virtually no difference in their support of coexistence or extermination, emphasizing the central place of feelings and beliefs about the species. This work echoes that conducted by Worcester and Boelens [52], who found that people who believe that urban wildlife is beneficial are less likely to see coyotes as problems and support coexistence. Finally, in a small survey study in Madison, Wisconsin, researchers found only approximately one-third of respondents had positive views of coyotes, with most identifying more risks than benefits from the species’ presence. These views were strongly correlated to respondents’ underlying ecological beliefs and, for some respondents, were also connected to the amount of attention they gave to wildlife news [53]. Together, these findings suggest that when urban residents have positive affective views of coyotes and believe they belong in the environment, they are more willing to support coexistence. While beliefs about coyotes come from a variety of places, ranging from underlying environmental views [53] to the type of community one lives in [54], one of the most well-established drivers of public perceptions of urban carnivores is the media [18,19,55,56]. 

### 2.4. News Coverage of Urban Coyotes

Coverage of coyotes as a social problem in urban environments is widespread [19]. Coyote attacks, in particular, prompt short-term increases in media coverage that not only describe the human trauma and individual problem animal but often present coyotes more broadly as pests and problems [17]. This short-term coverage negatively influences perceptions of coyotes, especially when they attack children [17]. Importantly, while extreme negative events like attacks on children are likely to spark justifiable concern or fear, the media often distorts neutral events in ways that amplify negative views. In their assessment of new stories about coyotes from the late 1990s and early 2000s, Alexander and Quinn [19] found that simple sightings and interactions were often described as attacks and that coyotes were frequently depicted as unnatural and invasive in urban environments. At that time, they and other scholars began calling for greater media literacy in the coverage of coyotes, pushing for a move away from unfriendly narratives to discussions of space sharing and coexistence [19]. This call was important because misinformation about coyotes is a significant barrier to effective and sustainable coyote management. When communities view coyotes as problematic or invasive, public attitudes often encourage their removal or extermination [17], and negative media attention about coyotes is a leading driver of fear and disinterest in coexistence in urban environments [57]. A decade after scholars began calling for the media to alter its narrative, there is some evidence of progress [58]. While the media still depicts coyotes as a risk in many areas, there is also a growing acknowledgment that coyotes belong in human environments. Looking at the media coverage of coyotes in Philadelphia, Pennsylvania, and Chino, California, Hunold and Lloro [58] found that, while people are not readily excited about coyotes in their communities, they recognize that habitat disruption has led to an increase in urban coyotes and that these animals belong. Changing coverage from pest and problem to coexistence has a real impact on public perceptions [58] and positively impacts support for urban coexistence efforts. As we think about urban environments’ long-term sustainability and ability to adapt and coexist in an ever-changing landscape, it is particularly important to evaluate whether there are more widespread changes in the media that transition framing away from the “coyote problem” and instead adopt language that is not entirely unfriendly.

### 2.5. Survival of the Friendliest and Coexistence with Urban Wildlife

Among evolutionary anthropologists and biologists, the theory of friendliness emerged [30,31,32] as an extension of the human self-domestication hypothesis [32]. The base concepts can be traced to Aristotle and his theory of friendship, which distinguishes friendship from goodness, pleasure, and utility [59,60]. The theory of friendliness focuses on the third component, utility, and specifically considers it in relation to human evolution. The argument is that humans evolved through cooperative networks that were utility driven. Hare and Woods [32] argue that, while much of evolutionary theory has been centered on Darwin’s survival of the fittest [61], the connotation of what fitness is has remained connected to physicality, suggesting that strength is the leading feature in survival, when, in actuality, Darwin was talking about the ability to reproduce. Hostility and chaos are not conducive to reproduction and, therefore, friendliness and cooperation may be more beneficial for survival than mere strength. In this context, friendliness refers to the demonstration of goodwill, showing warmth, and being kind. Hare and Woods [32] argue that we need to rethink human evolution to move away from a physical and combative perspective, instead centering cooperation and friendship as key mechanisms of our ability to meet both basic and complex needs. In this revision of evolution, Hare and Woods [32] assert that those who could cooperate for utility were more inclined to survival. Although this is a theory based on human evolution, they extend the idea to human–animal relationships, and the theory can be applied to evaluate some of the most important attitudinal shifts necessary to reduce cross-species conflict in urban environments.

Hare and Woods [32], themselves, began working on a cross-species theoretical application, starting their work with a description of the cognitive abilities of Hare’s dog Oreo. The vignette showcases how cross-species interactions around friendliness, even without a shared verbal language, dictate access and utility across species. They then use the domestication of the wolf as a central component of their theoretical presentation, explaining that the evolution of dogs demonstrates how survival of the friendliest equates to domestication and coexistence among species. They argue that when wolves decided to engage human settlements approximately 15,000 to 25,000 years ago, the friendliest among them bred and increasingly interacted with humans, who then set about changing their physiology and composition through breeding. Today, we know that friendliness on both sides has led to an unmatched, mutually beneficial relationship, and while we do not share verbal language with domesticated dogs, we each understand the others’ communicative gestures, something Hare and Woods [32] identify as a friendly strategy.

In terms of human and coyote encounters, friendliness between species is essential for both species’ safety and wellbeing. Negative depictions of coyotes and eradication methods have negative implications for human wellbeing—for example, their removal can lead to large increases in rodent populations—whereas sustainable management practices that promote coexistence and altered human behavior have been shown to decrease negative interactions and promote a unification between humans and animals. As noted previously, the single greatest predictor of support for human–coyote coexistence was individual affectual connection or perception of coyotes as friendly or part of the community [54]. If friendliness is such an important factor in human survival and coexistence among species, then how we depict species has implications for human and animal wellbeing. In this study, we evaluate how coyotes are depicted across the top newspapers in three major metropolitan areas, specifically focusing on friendliness. 

## 3. Materials and Methods

We assessed the coverage of coyotes in three major urban environments: Los Angeles, California; Seattle, Washington; and Boston, Massachusetts. These cities were chosen because they represent distinct US regions that have differing histories and relationships with coyotes. They also have important similarities that make them likely sites of evolving media coverage on coyotes: all cities have reported a significant increase in coyote sightings and interactions over the past decade, and all are considered green cities for their environmental policies and green spaces that include wildlife corridors. Data are derived from a content analysis of all newspaper articles from 2000 to 2022 discussing coyotes in the *Los Angeles Times*, *The Seattle Times*, and *The Boston Globe*. These are the major newspapers in the area that cover local, national, and international news. We used the following keywords to first identify articles: coyote, “coyote animal”, and “coyote attack”. All articles (N = 192) mentioning coyotes in the context of the city the newspaper represented were collected from 1 January 2000 to 31 December 2022 (23 years). Articles about coyotes that did not mention the city the newspaper represented were excluded. For instance, if the *Los Angeles Times* ran an article about coyotes in New Mexico without mentioning Los Angeles, the article was excluded. This resulted in 75 articles from the *Los Angeles Times*, 44 articles from *The Seattle Times*, and 73 articles from *The Boston Globe* (N = 192). All resulting articles were reviewed and line coded for the presentation of neutral facts, friendly language, unfriendly language, and mixed friendly and unfriendly language. Neutral facts consisted of information such as coyote range, food sources, and lifespan. Friendly language mentioned coyotes as friendly, community members, playful dogs, and/or promoted coexistence. Unfriendly language mentioned coyotes as a threat, suspicious, dangerous, problems, and/or promoted eradication. A second researcher coded all articles in NVivo. Incidents of mismatched coding were rare. In these cases, mismatched coding was discussed until 100 percent agreement was achieved. Based on the codes identified, articles were then classified into one of four categories: neutral facts, friendly, unfriendly, or mixed. Articles that only mentioned facts without friendly or unfriendly language were coded as neutral fact articles. Articles that did not mention eradication and had no mention of negative language (problem, dangerous, threat, etc.) were coded as friendly. Articles that only framed coyotes as problems, dangerous, and threats and discussed eradication were coded as unfriendly. Finally, articles that mentioned coexistence and eradication were coded as mixed. 

Some articles discussed incidents where coyotes attacked pets or people. These articles were coded in varied ways based on the coverage. Articles that discussed the attack factually but focused on coexistence were coded as friendly, even when they covered the trauma of the event and included quotes from distressed individuals. Similarly, articles that discussed incidents and focused on the eradication of packs were coded as unfriendly. Finally, articles that discussed these incidents and mentioned coexistence while also discussing the need to address and even eradicate an individual were listed as mixed friendly and nonfriendly. 

### Selected Cities

Los Angeles, with a population of 3.85 million people [62], is well known for human–coyote conflicts, which have been in the news for decades. Researchers believe coyotes have roamed the Los Angeles area since the Pleistocene, meaning that humans and coyotes have shared the land for virtually all of time. Because of this, multiple civilizations have interacted with coyotes in this area and have likely ascribed different cultural meanings to the animal. The Greater Los Angeles area is the ancestral homeland of the Tatavaim, Chumash, and Gabrieleño peoples [63]; although little is known about the ways these specific tribes viewed coyotes, many Indigenous peoples ascribe cultural, symbolic, and folkloric meaning to the animal [64]. Since colonization, human–coyote conflict has been a fairly consistent theme in the area, and the human population’s continued encroachment into coyotes’ natural habitat has led to heated debates about whether to eradicate or live with the species. The *Los Angeles Times* (*LA Times*) is the area’s largest metropolitan daily newspaper, with a daily readership of 1.4 million during the week and 2.5 million on Sundays. More than 22 million people visit the *LA Times* website each month. All *LA Times* articles discussing coyotes in the Greater Los Angeles area were retrieved and analyzed. 

Seattle, Washington, has a population of 783,919 [62]. Although there are no estimates of the number of coyotes that live in the metro area, the first coyotes settled in Seattle sometime between the 1920s and 1950s after their primary predator, wolves, were eradicated because of negative public perceptions of the species. Although a major US city, the municipality is also home to nearly 500 parks across 6441 acres that include extensive natural areas. As one of the most outdoorsy cities in the nation, Seattleites and coyotes alike use these park systems, creating multiple opportunities for human–coyote encounters. City residents are also known for their environmentalism. The combination of having a significant predator population, an environmentally focused populous, and significant coyote habitat has led to the creation of two carnivore projects focused on cohabitation and public education. *The Seattle Times* is the city’s primary newspaper with a readership of approximately half a million each day and 717,700 on Sundays. All of *The Seattle Times* articles discussing coyotes in the greater Seattle area were retrieved and analyzed.

Finally, Boston, Massachusetts, has a population of 654,777 [62]. Like Seattle, coyotes are a relatively new resident, who likely arrived in the late 1950s. The city, however, has traditionally had fewer interactions with coyotes. In addition to having less undeveloped open space and fewer park systems where coyotes might live (Boston manages 217 parks across 2346 acres), the city was densely developed and populated far earlier than Seattle, leading to fewer spaces that coyotes can inhabit. Yet, in recent years, sightings and frustration have increased as the species has been identified as a potential threat. *The Boston Globe* is the leading newspaper in this area, with a readership of approximately 244,190 print and digital subscriptions. All of *The Boston Globe* articles discussing coyotes in the Greater Boston area were retrieved and analyzed. 

## 4. Results

Table 1 provides a frequency table for all analyzed articles. Except for the theme “friendly”, all differences across cities are statistically significant at the <0.001 level based on chi-squared tests. City-specific results are discussed below. 

### 4.1. Coyote Coverage in Los Angeles, California

**Figure 1:**
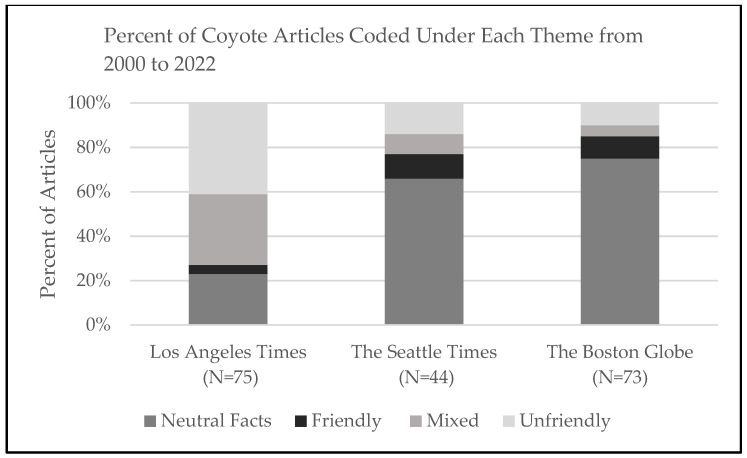
This graph depicts the percent of articles featured as neutral facts, friendly, mixed, and unfriendly across the three newspaper outlets.

The first *LA Times* coverage of coyotes in our timeframe occurred in 2001. This initial article is representative of most of the coverage over the next decade, depicting coyotes as an unfriendly, dangerous problem for humans, pets, and agriculture that needs to be eradicated. In one of the first articles in this series, coyotes were blamed for most cattle and calf deaths and were noted to be “less safe” than mountain lions and bobcats. Categorization of these articles as neutral, friendly, unfriendly, or mixed is provided in Figure 1. Of the 75 articles analyzed, 41 percent (31 articles) exclusively discussed coyotes as unfriendly problems to be eradicated. These articles clearly depict coyotes as unfriendly, a species that we cannot coexist with, and one that should be eliminated. For instance, a 2008 article discussed coyotes attacking and killing companion cats. The articles noted that “…coyotes present the danger, so they should be evicted”. Importantly, the unfriendly identification reflects how the article presents the appropriate response to coyotes rather than the precipitating event; if the 2008 article had covered cat attacks and discussed both coexistence measures, like keeping cats indoors and the possibility of euthanizing problem animals, it would have been coded as mixed. Comparatively, only four percent of articles (three articles) talked exclusively about coexistence. Thirty-two percent (24 articles) presented both sides of the debate. In discussing recent research on urban coyote management in Southern California, one article notes, “The reports are fueling an escalating war on Southern California’s urban coyotes, and exposing deep divisions between those who want to eradicate the animal and groups such as Project Coyote that call for peaceful coexistence”. Finally, 17 articles (23 percent) focused exclusively on general facts about sightings and scientific information and were, thus, coded as neutral. 

Most articles (74 percent) from 2000 to 2022 promoted eradication either exclusively or in combination with limited discussions of coexistence. Given the increasing evidence that eradicating coyote packs or reducing pack size creates problems for long-term management, one might expect the news media to decrease discussion of eradication and increase coverage of coexistence measures. However, there was no indication of an increase in exclusive discussions about coexistence over the last two decades. The last article exclusively discussing coexistence was in 2010. Since 2010, every article has focused on eradication (34 percent), taken a neutral stance (26 percent), or mentioned both eradication with coexistence (40 percent). One reason eradication appears to have dominated coverage over the last decade is that human–coyote encounters in Los Angeles have increasingly become a hot-button political issue. 

### 4.2. Coyote Coverage in Seattle, Washington

Seattle had the fewest coyote articles of the three analyzed cities (44 articles vs. 75 and 73), with the first coyote coverage in the time period appearing in 2005 when *The Seattle Times* presented readers with a community dilemma. An individual coyote had consumed a neighborhood cat and some small backyard livestock over the duration of a year, at which point the community began to question whether the coyote should be killed. This and three additional articles from 2005 to early 2008 painted coyotes as both a friend and foe, wild neighbor and nuisance, discussing the potential for both coexistence and eradication. In some ways, these early articles were an anomaly because, while human–coyote conflict is an ongoing concern for many Seattleites, the majority of articles (65.9 percent) discussed urban coyotes with neutral facts that paint the coyote as neither friend nor foe, see Figure 1. 

Only six articles (14 percent) discussed the removal or extermination of coyotes without discussing coexistence. Five of these articles were published in 2008 after a coyote took a cat and chased a dog in a major park and an increased number of people reported their pets being attacked or killed by coyotes, increases that likely stemmed from a moving or changing population. Interestingly, 25 percent (11 out of 44) of all articles published about coyotes in *The Seattle Times* were published in 2008, meaning that the media paid disproportionate attention to the issue that year and almost half of its coverage was unfriendly and advocated for extermination. These articles referenced the handful of attacks and used unfriendly language, such as the species being a danger, threat, hostile, unfriendly, and antisocial, to justify extermination. For example, one article explained, “…the coyote was showing aggressive tendencies towards people and animals” to justify shooting them (article from 2008). These articles demonstrate how negative events and fear can dramatically influence the media narrative. The increased incident reporting did not persist beyond 2008, though there is little reason to think coyote encounters have declined. Although it is beyond the scope of this paper, a key question emerges concerning the relationship between coverage and reporting: did reporting increase because of increased negative media coverage, or did increased media coverage reflect the consequences of higher incident reporting? Either way, *The Seattle Times* has not published articles exclusively focused on eradication since 2010. 

Five articles (11 percent) exclusively focused on coexistence. For instance, a 2017 article explains that “Coyotes have become our neighbors, whether we live in ruralized areas like Vashon or Bainbridge Island, the suburbs such as Normandy Park of right in Seattle”. In these instances, “friendly” language, such as friendly, neighborly, sociable, peaceful, and benign, were used to justify coexistence. In summary, during the period analyzed, discussions about coyotes remained largely neutral; however, when they appeared, eradication was justified through unfriendly depictions and coexistence was advocated for using friendly depictions. Neutrality and coexistence have entirely dominated the conversation since 2010. 

### 4.3. Coyote Coverage in Boston, Massachusetts

Coverage of human–coyote interactions did not appear in *The Boston Globe* until 2011, a decade after coverage began in the *Los Angeles Times* and about five years after it started in *The Seattle Times*. Just like the coverage in the other two newspapers, the first article in the series discussed the need to eradicate problem coyotes, but unlike the other two newspapers, the first article also emphasized that “residents are urged to remove food sources, such as pet food, fruit that falls from trees, garbage, bird feeders that attract small animals, and dead animals left lying around by cats”. This change makes a powerful statement in presenting the initial human and coyote interaction issue as a human behavior problem. Statistics around the presentation of friendly or unfriendly language as it applies to coyote eradication is markedly different in *The Boston Globe*. Only a handful (10 percent) of articles exclusively discuss eradicating coyotes, compared to 42 percent in the *Los Angeles Times* and 14 percent in *The Seattle Times*. 

Most discussion of euthanizing coyotes in *The Boston Globe* singles out an individual as the problem and not the entire population. For instance, in 2015, an article argues that trapping and relocation is essential when there are problem individuals causing issues for humans and pets. Similarly, in a 2016 article talking about problem coyotes terrorizing pets, a citizen was quoted as suggesting that part of the solution could be hunting problem coyotes. The man interviewed noted that he hunted the animals and tanned their hides, which he then gave away. Another example featured in a 2021 article does not advocate killing of coyotes but discusses why euthanizing particular animals might be necessary for human and pet safety. The article notes that, “Rangers regularly try to discourage coyotes who come into close contact with human-centered sources of food by hazing them with shouts and loud noises. And that sometimes works. But if a coyote learns where fishermen drop fish guts or where campers and picknickers gather, it becomes increasingly difficult to convince the animal to leave.” Unlike the other papers, this focus on the problem individuals does not vilify the species but acknowledges a complexity in coexistence that can result in the need to address challenges with one or more individuals.

Seven articles (10 percent) exclusively talk about coexistence and use statements that discuss the animals as being unnecessarily targeted. An article from 2015 notes that, “Misinformation, misunderstanding, and fear driven conflicts drive calls to trap and kill coyotes. Lethal control can disrupt group hierarchy, allowing more coyotes to reproduce, encouraging larger litter sizes due to decreased competition for resources, and increasing pup survival rates”. This article goes on to discuss how coyotes are relatively benign animals that are often friendly and that attacks largely occur because of human negligence. Finally, 55 articles (75 percent) were neutral articles that did not use friendly or unfriendly language to depict coyotes or discuss how they should be managed in urban areas. An example of a statement in this collection comes from a 2016 article, “Where I live, it’s best to experience nature in the daytime. Our woods are very active. Coyotes roam and black bears amble about. Certainly, anyone going into nature at night should be prepared with a plan of action should they encounter whatever wildlife might inhabit the territory”. This is a neutral statement that does not present wildlife as friendly or unfriendly, but as animals living in their homes. Importantly, while this quote reflects one aspect of coexistence—being prepared when humans go into nature—it is coded as neutral because it discusses humans going into nature, rather than wildlife entering urban habitats, does not attribute positive or negative traits to the species, and does not specifically advocate for an urban management approach. By far, *The Boston Globe* had the least entirely unfriendly articles about human and coyote interactions and conflicts. Overwhelmingly, articles depicted coyotes as neutral or positive animals in the urban environment and people as the key problem and solution to conflict. 

## 5. Discussion

The race to shape public sentiment around urban coyotes is on. Siemer and colleagues [20] note that there are windows of opportunity for conservationists to facilitate behavioral change and promote coexistence but that once negative (antifriendly) perceptions are engrained, it can be harder to change public attitudes. Public perceptions of urban wildlife and support for management strategies differ across urban areas, but it is essential that we understand how to encourage coexistence. Ecological research emphasizes the negative ecological and human impacts of eradication efforts and the importance of coexistence, and the media has a central role to play in encouraging support for this approach. Past studies suggest that media attention is the leading influence of affectual feelings towards coyotes [18,55,56] and that these feelings are central to views on coyote management, emphasizing how important it is for ecological health and conservation efforts that the media covers coexistence as a central strategy for managing coyotes. Our analysis of coyote media coverage in Los Angeles, Boston, and Seattle demonstrated the fact that there are significant differences in the way that the nations’ major newspapers discuss management efforts, with the *LA Times* emphasizing eradication efforts—either alone or in combination with the discussion of coexistence—at a higher rate than other papers and *The Boston Globe* presenting significantly more neutral coverage. Though not tested here, the differences in media coverage likely help account for the varied views on coyotes and support for coexistence across these cities. Scholars and practitioners understand that urban wildlife management plans must account for a community’s culture, norms, and perceptions of urban wildlife if they are to be successful [54], but the media and the way it primes and frames issues around coyotes not only reflects existing cultural norms and views, it also plays a significant role in shaping them. Thus, scholars, conservationists, and policymakers must be attuned to media coverage, public opinion, and policy impacts. Below we discuss the implications of our findings for management strategies in these independent urban environments. 

### 5.1. Implications of Findings for Los Angeles, CA

Los Angeles, CA, is considered an epicenter of human–coyote conflict. In fact, the problem has received so much attention that numerous environmental and governmental groups have been involved in addressing public sentiment and promoting human behavioral change. For instance, the LA Urban Coyote Project, which was started by researchers with the National Park Service at the Santa Monica Mountains National Recreation Area, was designed to gain a better understanding of coyote behaviors in the local areas and to educate agencies and the public on coexisting with coyotes in urban environments [65]. Although these programs are important and likely contribute to challenges and changes in public perception, the prevailing story about coyotes as dangerous, unfriendly invaders remains. This narrative is inconsistent with the bulk of the scientific evidence and may lead to greater human–coyote conflict, as it amplifies tensions and promotes ineffectual policy without helping the public understand the importance of coexistence or the need for human behavioral changes to minimize conflict. 

As with most municipalities, in Los Angeles coyotes have few legal rights. They are most frequently defined as pests, problem, or invaders. There is no limit on how many can be killed each year, though there are some restrictions on the methods used. For example, it is illegal to poison coyotes in the open or capture them in leg traps. It is also illegal to discharge a firearm in city limits, which likely reduces incidents of coyote killing. These prohibitions essentially comprise the entirety of the government regulation of coyote killing. California treats coyotes in the same way it treats rats and allows them to be trapped and euthanized. It is illegal to transplant a coyote, so the only option once an animal is trapped is to kill them, and there are several companies that perform this service with or without cause. For instance, Los Angeles Coyote Trapping & Removal Experts [66] is a company that has been in business for over 20 years specializing in removing and euthanizing problem wildlife with a particular focus on coyotes. In fact, the dominant form of wildlife “pest control” in major cities is private companies rather than conservation organizations, meaning that perceived aggressive or suspicious behavior is often lethal for coyotes [67]. 

Despite the lack of regulation, the California Department of Fish and Wildlife promotes coexistence in line with scientific evidence. The state notes that coyotes are an important part of the ecosystem, keeping rodents under control and serving as scavengers. However, the state uses the term “hazing” in advising people to engage in a process of instilling fear in coyotes to scare them away and reduce conflict. This includes making noise, maintaining eye contact, throwing rocks or sticks, stomping feet, making shaking noises, and using airhorns or water guns [68]. These are sound suggestions, but the use of hazing has a negative connotation often associated with teasing, mocking, heckling, bothering, or annoying with the end goal of making the recipient feel uncomfortable or scared. Such language not only places the human on the offensive but also centralizes the animal as an invader that deserves to be terrorized. Beyond hazing, the state also offers a list of individual behavioral changes that can be made to limit coyotes becoming a problem [68]. 

Social, cultural, and racial depictions of coyotes cannot be decoupled from the realities of how management unfolds across the United States [69]. Beyond media depictions, some scholars argue that the use of “coyote” as a term for those who smuggle people across the Mexican–United States border has furthered negative sentiment about the animal, especially in states bordering Mexico [67]. Social and mass media depictions of coyotes as lawless outlaws, interlopers, criminals, immigrants, and gang members, especially among southern border states, are infused with racist connotations around movement and predation that have historically been applied to people of color and especially to those of Latin American decent [67,70]. For some species—like the coyote in specific locations—racism has become infused with urban pest control, transitioning the coyote as an animal as a symbol of evolving social and cultural dynamics around who has access to space and free movement [71].

Given the lengthy history of the human–coyote conflict in Los Angeles, recent research touting coexistence and the broad promotion of changes in human behavior as a solution, we expected to see a marked increase in the coverage of coexistence and a reduction in narratives that paint coyotes as unfriendly invaders over time. However, despite scientists and the state government’s focus on coexistence, depictions of the “coyote problem” in the *Los Angeles Times* appear largely unchanged. Although there were slight increases in articles mentioning the need for behavioral changes among humans, all articles post-2010 included discussion of coyotes being problems that should be removed and/or eradicated. These articles furthered the narrative of the coyote as invader, unwanted, and dangerous. Disdain over and conflict with coyotes is likely to continue unless the media narrative shifts to be one about inclusion and coexistence that includes a shift towards friendly language that emphasizes the species’ contributions. Misinformation and negative depictions by the media are likely influencing general perceptions in ways that undermine evidence-based policy and the efforts of environmental and conservation groups. Given negative sentiments and the connection between coyotes and other social issues, particularly around race and immigration, envisioning new possibilities for coexistence may be challenging. The mismatch in presenting the problem relative to science may be exactly what Siemer and colleagues [20] articulate in asserting that, once an issue is established as a problem and formulated around negative depictions, negative perceptions develop that reinforce the narrative even if the science has changed. 

Despite this, research on other predators offers hope that change is possible. For example, the media has been shown to generate policy stories around wolf reintroduction in Yellowstone, not just reflecting existing policy narratives and views but shaping them [72]. In Australia, framing shark bites in ways that help explain the behavior and remove intent has been shown to reduce fear and limit support for lethal mitigation [73,74]. And, in Brazil, residents are more likely to support jaguar conservation when they trust and understand the management agency [13]. This research suggests that media can play a significant role in shaping predator policy views and the causal stories that underly them. Framing animals in friendly terms and in ways that support coexistence can help overcome culturally imbedded fear and decrease support for extermination, while providing fair and detailed coverage of how predators are managed and by whom can help residents support coexistence, even with animals that have long been viewed as problems. In other words, the *Los Angeles Times* cannot control whether there are problem coyotes, but they can control the explanations they provide for their behavior and the management strategies they discuss as a response; increasingly incorporating friendly language, discussions of the human causes of conflict, explanations of coexistence strategies, and coverage of management efforts may well improve public opinion of coyotes and willingness to cohabitate with them. 

### 5.2. Implications of Findings for Seattle, WA

In Seattle, WA, media coverage of human–coyote interaction and conflict has changed over the past two decades, likely altering public perceptions, human behavior, and conflict. Media coverage started with a moral dilemma about killing a “rogue” coyote who appeared aggressive in eating a cat and small livestock transitioned to depicting coyotes largely as unfriendly, and this is transitioning again to reflect the scientific reality that coexistence is likely the only viable option, since the coyote range is expanding, and trapping and removal often exacerbate conflicts. Since 2010, news reports of coyote sightings and human–coyote encounters in *The Seattle Times* have largely been neutral or benign, but there has been increasing coverage of the human behavioral changes required to reduce conflict and friendly language used to discuss coexistence. Transitions of media coverage in *The Seattle Times* are in line with the state’s position on human–coyote interactions. 

The Washington Department of Fish and Wildlife [39] notes that it is not necessary or realistic to remove all coyotes from any area, urban or otherwise, and that it is illegal to trap and relocate coyotes, as this practice is often ineffective and inhumane. The state argues that the best approach is coexistence, noting that attacks are rare and that only two people have ever been bitten in the state, both of which likely occurred because the animals were being fed by humans. The state makes a number of recommendations to reduce human–coyote conflict, all of which center on human behavioral changes, such as do not leave small children unattended, never feed coyotes, secure garbage, feed dogs and cats indoors, do not feed feral cats, watch dogs and cats outdoors, especially during dusk, build coyote-proof structures, enclose chickens and other birds in secure pens, secure livestock indoors, and remove and bury dead animals [39]. Variations of these suggestions appeared in all of *The Seattle Times* articles post-2010. There are a number of organizations in addition to the Washington Department of Fish and Wildlife that have worked to educate the media and public in ways that establish coexistence with coyotes as a norm among the public. Among others, these include the University of Washington and the Seattle Coyote Study [75], which is funded through the National Science Foundation, Seattle University, and Woodland Park Zoo’s Seattle Urban Carnivore Project to track coyote movements and human interactions [4], and the Carnivore Spotter [76] to document sightings. These government, academic, nonprofit, and conservation efforts facilitate real work on the ground, and the media has served an important role in educating the public about both the work and recommendations.

Together, this suggests that media coverage and evolving science regarding coyotes in the Seattle area are in alignment. More specifically, the norms and behaviors outlined by the state, which are based in science and communicated through the media, will not only benefit humans, but support the wellbeing of urban wildlife broadly, which includes animals such as bobcats, raccoons, bats, and squirrels in this area. 

### 5.3. Implications of Findings for Boston, WA

Media coverage of coyotes in Boston, MA, has not changed much over the past decade, with the first and most of the subsequent articles focusing on either neutral content or coexistence in ways that align with the scientific consensus. In the *Los Angeles Times*, readers are exposed to coyotes as invaders, wreaking havoc on the community. In contrast, the narrative in *The Boston Globe* is more like the later years of *The Seattle Times*, minimizing negative portrayals of the entire species and instead focusing on individual problem animals and how humans have contributed to or created these issues. Boston is unique in that media coverage of urban coyotes is relatively new, only surfacing in *The Boston Globe* in 2011, which was also when there began to be greater awareness that lethal removal methods are ineffective and that coexistence is the only strategy to promote wellbeing among species. It is likely that the city’s limited history with coyotes delayed coverage in ways that allowed its major paper to start its coverage with content that aligns with current scientific consensus, framing the animal with more neutral or friendly language and priming residents for coexistence measures without problematizing the entire species. 

Like CA and WA, the state of MA is clear about the need to coexist with coyotes, but there are a few differences in terms of protections. In MA, coyotes are referred to as a shy and elusive furbearing species by the Division of Fishers and Wildlife [60]. The classification of a furbearing resource means that their population is managed and that a regulated hunting and trapping season has been established that usually runs from mid-October to mid-March (5 months) [77]. Although there is no limit on coyotes killed, all hunted and retrieved coyotes must be reported. Coyote trapping is also allowed. Like the LA and Seattle, Boston allows property owners to hire problem animal control agents (PAC) if coyotes are damaging property or posing a threat to safety. However, PAC agents can only address coyote-related issues if they have completed a Mass Wildlife training and have a certification to deal with coyotes [78]. This is unique to the state. Much like other states, Massachusetts is clear about their position on coexistence, offering a wealth of information about how humans can reduce human–coyote conflict. 

It is beyond the scope of this paper to assess whether more friendly media coverage of coyotes in Boston has contributed to better management strategies or increased support for coexistence, but evidence from other species and places suggests this is likely the case. Further, we feel confident asserting that, because *The Boston Globe* has avoided vilifying the species and promoting widespread extermination efforts, the paper is, at a minimum, unlikely to have a negative impact on efforts to promote coexistence measures. In fact, recent coverage suggests it is likely helping raise awareness about the importance of coexistence. For instance, in light of increased coyote sightings, in 2022, the MSPCA, which is one of the nation’s first animal advocacy organizations, launched a campaign to encourage coexistence. The campaign was headed by MSPCA’s Elizabeth Magner, PhD, who has historically refused to assert that coyotes are a problem, instead referring to the issue as a “non”-problem while advocating for human behavior adjustments to protect humans and support coyotes in their ecosystem functions [79]. The effort has received significant media attention that centralizes coyotes as part of the community, uses friendly language, and provides the public with key information about coexistence.

## 6. Conclusions

Friendliness is a pillar of coexistence [30,31,32]. As Hare and Woods suggest, humans and domestic dogs coevolved through collaboration and cooperation; should either species have decided on conflict as the leading strategy, one or both would not have fared as well [32]. Over the past decades, coyotes (a type of wild dog) have expanded across North and Central America and into all contiguous US states, except Alaska. Much of this expansion has been into urban areas, which has prompted fears of coyotes attacking companion animals and humans. Discussions of eradication were prominent for decades before scientific consensus coalesced around coexistence as the only feasible solution. Recent research suggests that effective coyote management strategies should be localized by first assessing coyote behavior, human resident behavior, area knowledge about coyotes, and public perceptions of the species [5,80]. A key omission in these and other studies is the impact that social and mass media has had on the public’s perception of wildlife.

Media is a powerful tool when it comes to human–wildlife interactions and conflict. Negative depictions of conflicts that are caused by humans but blamed on the animal—such as feeding wild animals and prompting an attack—can rapidly change public perception of wildlife and reduce support for coexistence [81,82,83]. We built on the existing literature and extended the theory of friendliness by evaluating media depictions of coyotes in three urban areas: Los Angeles, California; Seattle, Washington; and Boston, Massachusetts. We used the theory of friendliness to assess the unfriendly and friendly language used to justify the coexistence or eradication of coyotes over the past 23 years (2000–2022). We then assessed how the three selected urban areas differed in their coverage and approach to coyote management. 

Media depictions vary considerably across localities but across the three newspapers, friendly language correlated with discussions of coexistence, while unfriendly language correlated with negative portrayals and discussions of lethal management. Over the past 23 years, a significant amount of coverage has also provided neutral, fact-based information about the species. Although knowledge about a species has a less direct effect on public opinion and support for management strategies than affective feelings [14], it plays a key role in shaping affective feelings, with sound scientific understanding generally being associated with more positive feelings [84]. While coverage in Los Angeles, CA, remained largely negative across the entire period, coverage in Seattle, WA, saw a reduction in negative depictions and associated talks of eradication of individuals, packs, and species over time, and Boston, MA, virtually never engaged the eradication conversation. In areas like Los Angeles, CA, where coyotes have been depicted as a prevalent and enduring problem for decades, public perception, management, and coexistence may be slow to change, making media literacy around coyote science essential. 

There are several limitations to our study. First, though the survival of the friendliest theory is useful in helping us think about why framing and friendliness language matter, the theory focuses on utility in a way that may be culturally contingent. Utility is a culturally specific way of viewing human–animal relationships that may not resonate with all cultures, particularly those of Indigenous peoples [85]. Consequently, though there is evidence that friendly media framing is likely to influence perceptions across cultures, the specific connection to utility—for example, the coyote’s importance for rodent management—may not be the best approach in all cultures. This question should be tested and explored in cross-cultural ways in future research. Second, while there is strong evidence that human–dog relationships have played an essential role in human evolution and development across cultures [86], modern societies’ views of canines vary considerably and should be considered in evaluating the influence of friendly language on residents’ views and behaviors towards canines. Although we began to see this in our study by evaluating three cities with distinct cultural and historic views of coyotes, we were unable to provide international comparisons or to evaluate how individuals from different cultures in a single location respond to friendly language; future work should explore this question. Third, though the coyote’s range extends across North and Central America, we focused exclusively on three cities in the United States; because of this, while the study adds to a growing international literature examining media coverage of predators, specific findings may not be applicable outside of the US, particularly since wildlife values vary considerably across countries and help shape views of animal policy [85]. Finally, although media coverage has direct implications for public opinion, we did not measure this and instead used past research to draw out the potential implications of news coverage. 

There are several areas for future work. Our analysis demonstrated significant differences in the type and scientific soundness of coverage across urban environments. Although past work suggests these differences likely help explain the distinct public opinion and policy debates across these localities, future work should investigate this directly. For all cities considering strategies to improve human–coyote coexistence, news coverage of coyotes should be analyzed and used to inform management strategies, with the specific acknowledgement that widespread antifriendly coverage may not only mean the public is less familiar with coexistence measures but also less primed to support these policies. All strategies should consider the influence of mass and social media and start by addressing literacy gaps among media outlets in understanding and presenting coyote science. Educating media outlets about coyote science and how to present stories about coyotes to promote positive outcomes may more beneficial than simply educating the public, whose opinions about management are most strongly shaped by affective feelings. As it stands, it appears that negative media portrayals that counter scientific consensus and promote sustained conflict or eradication likely make it harder for advocacy groups and state departments to promote coexistence. Across all cities interested in management strategies, studies to assess public perception and associated behaviors to avoid human–coyote conflict are needed. These studies should be triangulated with an assessment of media coverage to assess causal inference.

Ultimately, to promote coexistence, reframing messages about coyotes from problem and pest to important cohabitant is likely key in limiting conflict. Family focused messages highlighting the benefits the species brings and educating the public about coyote safety are most effective in influencing prosocial and pro-coyote behaviors [6]. When family is placed at the center of a message, viewers are more likely to feel that the role they play in human and coyote safety is important. These messages do not problematize the existence of coyotes but promote collective engagement with humans centered on protecting both species through targeted behavior, given that the greatest predictor or support for coexistence is positive perception of coyotes as embedded within one’s community [42]. Positive public attitudes regarding coyotes and coexistence cannot be obtained if media portrayals continue to counter scientific consensus and push an unfriendly narrative. In terms of urban human–coyote interactions, friendliness is key to coexistence. 

## Figures and Tables

**Table 1:** Frequency of themes across cities showing significant differences in coverage.

	Frequency of Themes Across Cities		
	Los Angeles	Seattle	Boston
Neutral ***	17	29	55
Friendly	3	5	7
Unfriendly ***	31	6	7
Mixed ***	24	4	4
Total	75	44	73

*** Differences across cities are statistically significant at the <0.001 level.

## Data Availability

Data presented in this study are available upon request from the corresponding author. Data are not publicly available currently because of ongoing research projects.
